# Enhancing Interest in Smoking Cessation Programs With Nudge-Incorporated Flyers: A Randomized Controlled Trial Among Occupational Health Staff and Workers in Japan

**DOI:** 10.7759/cureus.64756

**Published:** 2024-07-17

**Authors:** Masaki Takebayashi, Yudai Kaneda, Mayumi Ouchi, Takao Sensui, Kazushi Yasaka, Mira Namba, Kurenai Takebayashi, Hirohide Shibutani, Tatsuya Koyama

**Affiliations:** 1 Sociology, Aomori University, Aomori, JPN; 2 Health Sciences, Aomori University of Health and Welfare, Aomori, JPN; 3 Medicine, Hokkaido University, Sapporo, JPN; 4 Solutions, Sangyoui Inc., Tokyo, JPN; 5 Smoking Cessation Support, Linkage Inc., Tokyo, JPN; 6 Medicine, Keio University, Tokyo, JPN; 7 Health and Wellness Planning, Olympus Co., Tokyo, JPN; 8 Human Life Sciences, Mimasaka University, Okayama, JPN

**Keywords:** smoking cessation program, randomized controlled trial (rct), japan, nudge, occupational health

## Abstract

Purpose

This study aimed to investigate the willingness to use and the application interest toward a smoking cessation program flyer among occupational health staff and smokers, utilizing a nudge approach.

Methods

A control group (typical flyer) and a nudge group (flyer improved according to the Easy, Attractive, Social, Timely (EAST) framework from the control flyer) were established. Occupational health staff and workers with a desire to quit smoking were randomly divided into two groups, and a web survey was conducted.

Results

Among occupational health staff, the nudge group flyers received significantly higher evaluations with desires "to apply" (control group: 1.7±0.7 vs. nudge group: 3.7±1.2: 5-point scale) and "to recommend to colleagues in the same profession" (control group: 1.7±2.4 vs. nudge group: 6.6±2.4: 11-point scale), and the reading completion rates were 7.0% for the control group vs. 70.7% for the nudge group (p<0.001). Although there was no significant difference in smokers' willingness "to apply" (control group: 2.9±1.2 vs. nudge group: 3.1±1.2: 5-point scale; p=0.388), the nudge group flyer was significantly more likely to be "want to recommend to other smokers" (control group: 4.9±2.4 vs. nudge group: 5.5±2.4: 11-point scale; p=0.032), with reading completion rates of 73.1% for the control group and 87.4% for the nudge group (p=0.001).

Conclusion

Typical flyers were not preferred by occupational health staff and may not have been effectively promoted to workers wishing to quit smoking. This study suggests that the combination of the EAST nudges could potentially increase the appeal to occupational health staff. To enhance the application interest among workers wishing to quit smoking, introducing other methods such as incentives might be necessary.

## Introduction

Smoking is a significant contributor to noncommunicable diseases, with approximately one person dying every four seconds due to tobacco [1]. While noncommunicable diseases are often viewed as a problem in low- and middle-income countries, smoking is also a serious issue in developed countries [2]. In Japan, smoking causes 130,000 deaths annually [3]. Despite the Japanese government's health promotion strategy, "Health Japan 21 (second term)," setting a target smoking rate of 12% (combined for men and women) for 2022, the actual rate remained at 16.7% (27.1% for men and 7.6% for women) [4]. By age group, the smoking rate for men is 25.5% for those in their 20s and remains steady at around 30% from their 30s to 60s but dramatically decreases to 15.1% among those in their 70s. Similarly, among women, the smoking rate is around 7% in their 20s and 30s, around 10% in their 40s and 50s, and 8.6% in their 60s, but drops to 3.0% among those in their 70s [5]. Thus, smoking is primarily a challenge among the working-age population in Japan.

Many smokers have a desire to quit smoking [5-7]. In response to this, several smoking cessation programs targeting the working-age population have been developed [8-10]. However, not all smokers with a desire to quit smoking are enrolling in these programs. One reason smokers desiring cessation may not quit smoking despite their desire is because of cognitive bias. For example, smokers tend to have a strong present bias [11,12]. Smoke cessation involves typical intertemporal choices, which are decisions with consequences that play out over time [13]. Individuals with a strong present bias tend to procrastinate implementation [14]. It is expected that promoting smoking cessation in line with smokers' cognitive biases can help avoid procrastination in enrollment in programs.

One approach to addressing cognitive biases is through nudges. A nudge is defined as "any aspect of the choice architecture that alters people's behavior in a predictable way without forbidding any options or significantly changing their economic incentives [15]." It is important to use frameworks like the EAST (Easy, Attractive, Social, Timely) when designing nudges [16]. The Japanese government advocates for the use of the EAST nudges in healthcare [17]. While the EAST can be also important in smoking cessation, we were unable to find reports of smoking cessation programs designed with the EAST nudges. Additionally, even if smoking cessation programs designed with the EAST nudges are developed, they may not be promoted to workers unless adopted by occupational health staff, such as company doctors or occupational health nurses. Therefore, smoking cessation programs need to be designed with promotions that are acceptable to both occupational health staff and workers desiring smoking cessation in order to be widely adopted. This study aims to investigate the evaluation of both occupational health staff and workers desiring smoking cessation regarding the design of smoking cessation program flyers with the EAST nudges. The hypothesis is that designing flyers with the EAST nudges will lead to favorable reception by both occupational health staff and workers desiring smoking cessation.

## Materials and methods

Research design

This study employed a parallel-group randomized controlled trial with an intention-to-treat analysis according to the CONSORT statement [18].

Participants

This study consisted of two phases: Phase 1 was for the first customers, occupational health staff, and Phase 2 was for the second customers, workers desiring smoking cessation. 

Occupational Health Staff (Phase 1)

Initially, the recruitment of occupational health staff through an internet survey company was planned. However, it proved difficult to attract many staff in a short period of time through the company. Consequently, recruitment was conducted among individuals who applied to participate in a smoking cessation promotion seminar for occupational health staff, which was organized by the company associated with the third author. Those who applied voluntarily were randomly assigned to the control group and the nudge group. Exclusion criteria were those under 20 years old and unwillingness to participate in the survey. No financial incentives were provided to respondents.

Workers Desiring Smoking Cessation (Phase 2)

This group consisted of current smokers who voluntarily applied through an internet survey company, Macromill, Inc. (Tokyo). Participants were randomly assigned to two groups through Macromill, Inc.'s system. Exclusion criteria were those with unwillingness to participate in the survey, lack of desire to quit smoking, under 20 years old, over 70 years old, and being an occupational health staff member. Frameworks were set up to ensure an equal distribution of gender and age among applicants. After the survey, Macromill, Inc. awarded predetermined points based on the number of responses.

Interventions

Two types of promotion flyers were created for a smoking cessation program for corporate employees, jointly conducted by Sangyoui, Inc. (Tokyo) and Linkage, Inc. (Tokyo). This program primarily involved using nicotine gum as a substitute for smoking during cravings and receiving smoking cessation information via social networking services. From February to March 2024, recruitment, interventions, and web-based surveys were conducted. Due to the nature of the study, double-blinding was not performed.

Control Group

**Figure 1:**
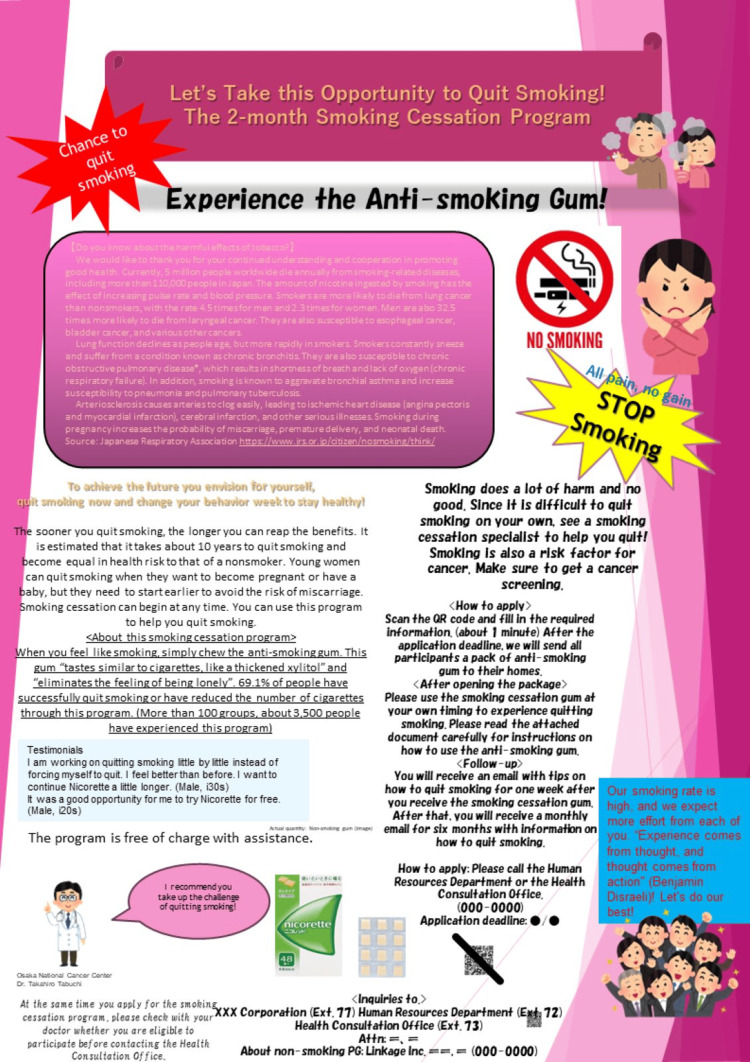
Control group’s flyer Image Credits: Masaki Takebayashi

The control group’s flyer is shown in Figure 1. We selected and modified a typical smoking cessation program flyer used by our client companies. This flyer provided detailed information about the smoking hazards and the necessity of smoking cessation, along with a description of the program.

Nudge Group

We identified issues with the control group’s flyer from the perspective of EAST nudges. As a result, inhibitory factors were estimated such as “difficulty understanding how to apply for the smoking cessation program” (lack of Easy nudge) for occupational health staff, and “excessive amount of information” (lack of Easy nudge), “lack of attractiveness” (lack of Attractive nudge), “lack of feeling that others are applying for the program “ (lack of Social nudge) for smokers, which could result in a tendency to postpone enrollment (lack of Timely nudge).

To address these inhibitory factors, we deleted information that most people would already know (Easy nudge). We used a title that said, "How about trying our anti-smoking gum for 2 months?" which induced smokers’ feeling that "This seems like something I could do myself" (Easy nudge). Additionally, we focused on the habit of busy workers to immediately look at titles and four-panel comics when reading newspapers. The four-panel comic was designed to be eye-catching, using a large space (Attractive nudge). Furthermore, we used expressions suggesting that many smokers are using the program (Social nudge) and prominently displayed a recommendation comment with a photo from a well-known expert (Attractive nudge). The design also allowed for immediate application and prevented procrastination (Timely nudge).

**Figure 2:**
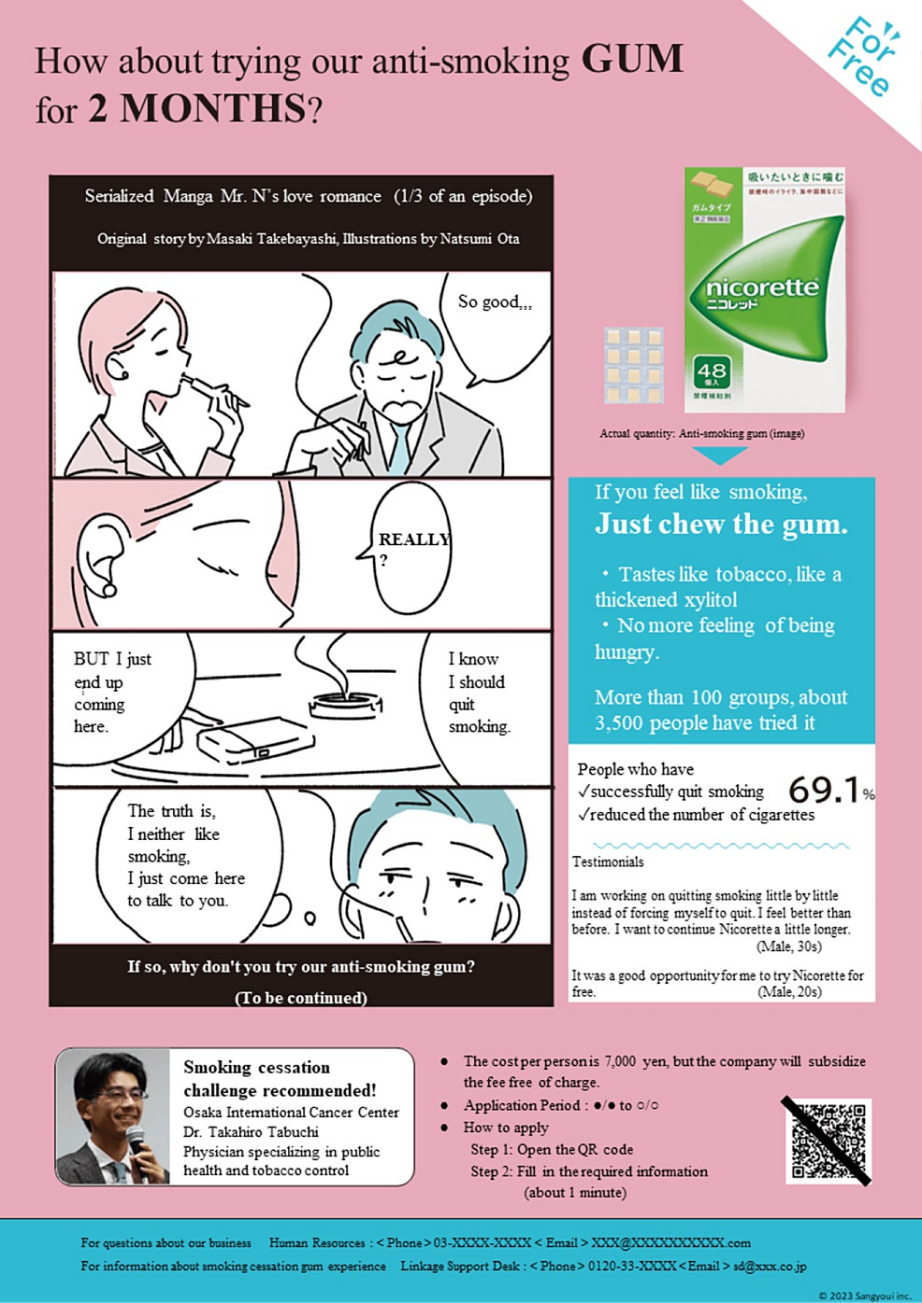
Nudge group's flyer The comic was the first part of a three-part series. Image Credits: Masaki Takebayashi

In designing these nudges, we received advice from external behavioral economics researchers beforehand and sought opinions from 21 occupational health staff and 21 smokers as a preliminary study. Figure 2 shows the finalized Nudge Group flyer.

Outcomes

The primary outcomes were assessed using a 5-point scale ranging from "strongly disagree" to "strongly agree" for the question "Would you like to use this flyer?" (for occupational health staff) and "Did this flyer increase your willingness to apply?" (for workers desiring smoking cessation). The question "Would you recommend this flyer?" was assessed on a scale of 0 to 10, following previous research [19]. Additionally, respondents were asked "Did you read this flyer to the end?" with the options "Yes/No."

Secondary outcomes included questions such as "Was it easy to read?" and "Did you feel it was easy to apply?" (corresponding to the Easy nudge), "Did it catch your eye?" (corresponding to the Attractive nudge), "Did you feel that many people would apply?" (corresponding to the Social nudge), and "Did you feel like reading it immediately?" (corresponding to the Timely nudge). These questions were designed in accordance with those used in previous research [20]. A free-text comments section was also provided.

Ethical considerations

The study was approved by the Research Ethics Committee of the Faculty of Sociology, Aomori University (approval no. 07-2023). The methods were conducted in accordance with the Declaration of Helsinki and Good Clinical Practice guidelines. Occupational health staff and workers desiring smoking cessation were informed of the voluntary nature of participation, privacy protection, and that there would be no disadvantages if they chose to withdraw midway. Participants were instructed to mark a checkbox indicating their consent before answering. After the study was completed, both flyers were published for all participants.

Statistical analyses

The continuous data were analyzed by t-test and the categorical data were analyzed by Fisher’s exact test or the chi-square test. SPSS version 28 (IBM, Tokyo) was used for analysis, with a significance level of 5% (two-tailed).

## Results

**Figure 3:**
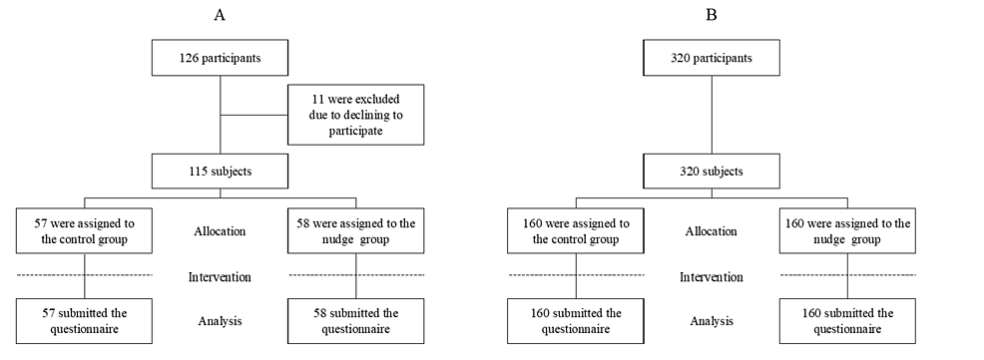
Trial profile A represents the flow diagram of the occupational health staff (Phase 1), and B depicts that of workers desiring smoking cessation (Phase 2).

In phase 1, of the 136 occupational health staff participants in the seminar, 115 agreed to participate in the study. Subsequently, 57 were assigned to the control group and 58 to the nudge group, with all responses included in the analysis (Figure 3A). Additionally, in phase 2, all 320 workers desiring smoking cessation who applied through Macromill, Inc. consented to participate in the study. These participants were divided, with 160 assigned to the control group and 160 to the nudge group, and all responses were included in the analysis (Figure 3B).

There were no significant differences observed in basic attributes in both phases (Table 1).

**Table 1:** Basic characteristics of the participants The significance level was defined as P<0.05 (two-tailed). ^a^The comparison was made using chi-square tests or Fisher's exact test. ^b^The study targeted only workers desiring smoking cessation. Based on the transtheoretical model, individuals in the precontemplation stage were those who "want to quit smoking but not within the next 6 months," those in the contemplation stage were those who "want to quit smoking within the next 6 months but not within the next month," and those in the preparation stage were those who "want to quit smoking within the next month" [21]. ^c^The comparison was made using t-tests.

Items	Occupational Health Staff (Phase 1)					Workers Desiring Smoking Cessation (Phase 2)					
	Control Group (n=57)		Nudge Group（n=58）		P-value	Control Group (n=160)		Nudge Group (n=160)		P-value	
	n	%	n	%		n	%, SD	n	%, SD		
Sex	-	-	-	-	0.675	-	-	-	-		1.000
Men	10	17.5%	13	22.4%	-	80	50.0%	80	50.0%		-
Women	47	82.5%	45	77.6%	-	80	50.0%	80	50.0%		-
Age^a^	-	-	-	-	0.622	-	-	-	-		1.000
20s	3	5.3%	6	10.3%	-	32	20.0%	32	20.0%		-
30s	16	28.1%	14	24.1%	-	32	20.0%	32	20.0%		-
40s	22	38.6%	22	37.9%	-	32	20.0%	32	20.0%		-
50s	11	19.3%	14	24.1%	-	32	20.0%	32	20.0%		-
60s	5	8.8%	2	3.4%	-	32	20.0%	32	20.0%		-
Behavior change stage^a,b^	-	-	-	-	-	-	-	-	-		0.758
Precontemplation	-	-	-	-	-	93	58.1%	89	55.6%		-
Contemplation	-	-	-	-	-	42	26.3%	41	25.6%		-
Preparation	-	-	-	-	-	25	15.6%	30	18.8%		-
Number of cigarettes (/day)^c^	-	-	-	-	-	13.3	±9.8	12.9	±8.0		0.658
History of Smoking (years)^c^	-	-	-	-	-	20.8	±14.1	20.5	±12.9		0.852

The outcomes are presented in Table 2. In Phase 1, the nudge group scored significantly higher in all items. In Phase 2, the nudge group scored significantly higher on five items: "Want to recommend it to others," ”Read it to the end,” "Easy to read," "Easy to apply," and "Feel that many smokers would apply."

**Table 2:** Outcomes The analysis was conducted excluding missing values. ^a^The ratings were evaluated on a 5-point scale from "strongly disagree: 1" to "strongly agree: 5," and compared using t-tests. ^b^The degree of willingness to recommend was evaluated on an 11-point scale from 0 to 10, and compared using t-tests.

Items	Occupational Health Staff (Phase 1)					Workers Desiring Smoking Cessation (Phase 2)				
	Control Group (n=57)		Nudge Group（n=58）		P-value^c^	Control Group (n=160)		Nudge Group (n=160)		P-value^c^
	Average	SD	Average	SD		Average	SD	Average	SD	
Interest in using it/increase in willingness to apply^a^	1.7	±0.7	3.7	±1.2	<0.001	2.9	±1.2	3.1	±1.2	0.388
Want to recommend it to others^b^	1.7	±2.4	6.6	±2.4	<0.001	4.9	±2.4	5.5	±2.4	0.032
Easy to read^a^ (Easy nudge)	1.4	±0.6	3.8	±1.2	<0.001	2.8	±1.2	3.4	±1.1	<0.001
Easy to apply^a^ (Easy nudge)	1.6	±0.8	3.4	±1.1	<0.001	2.9	±1.0	3.3	±1.0	0.001
Catching attention^a^ (Attractive nudge)	2.6	±1.2	3.5	±1.3	<0.001	3.0	±1.1	3.2	±1.1	0.093
Feeling that many smokers would apply^a^ (Social nudge)	1.7	±1.0	3.2	±1.1	<0.001	2.6	±1.0	2.9	±1.1	0.016
Motivation to read it immediately^a^ (Timely nudge)	1.6	±0.8	2.9	±0.8	<0.001	2.7	±1.1	2.9	±1.1	0.078

Regarding free-text comments, 35 comments were obtained from the control group and nine from the nudge group in Phase 1, and 66 were obtained from the control group and 36 from the nudge group in Phase 2. These were categorized as "positive" or "negative" based on their content (Table 3).

**Table 3:** Free-text comments

Occupational Health Staff (Phase 1)		Workers Desiring Smoking Cessation (Phase 2)	
Control Group	Nudge Group	Control Group	Nudge Group
(Positive: 0 comments) -	(Positive: 4 comments) It is easy to read because there are not many words. It is easy to read because there are not too many words. It is easier to attract my interest than a flyer with only words. It contains necessary information and is easy to understand.	(Positive: 6 comments) Impactful (3 comments); easy to understand (3 comments)	(Positive: 19 comments) The manga made it easy to read (6 comments). It made me feel like trying to quit smoking (4 comments). The design is good and easy to read (9 comments)
(Negative: 35 comments) The text is too small and there is too much information (33 comments). I don't like the illustrations. Overall, it is unpleasant.	(Negative: 5 comments) The text is too small. The cartoon should be placed on the right side for easier reading. I don't like the coloring. I thought it was advertising. I don't read manga unless I have time.	(Negative: 60 comments) Too much information (40 comments); lack of impression (15 comments); lacks the motivation to quit smoking (5 comments)	(Negative: 17 comments) I want a more detailed explanation (6 comments); not very appealing (11 comments)

## Discussion

Occupational health staff rated the nudge group's flyer significantly higher in all items, and workers desiring smoking cessation showed four significantly higher items. Therefore, while the hypothesis of this study, "the use of nudges in smoking cessation program flyers would be favorably accepted by both occupational health staff and workers desiring smoking cessation," was supported for occupational health staff, it was partially supported for workers desiring smoking cessation.

In the control group, the overall evaluation was that the flyer did "not feel like applying, not recommendable, and not read until the end." In the control group, out of 57 respondents, 35 wrote free-text comments, with 33 of them stating, "Too much information." This suggests that many occupational health staff felt cognitive ease being compromised by information overload. Without cognitive ease, people are more likely to be vigilant and suspicious, so it is speculated that occupational health staff did not have a strong motivation to actively read the flyer in the control group [22]. The flyer in the control group, based on existing flyers, indicated that there had been low satisfaction promotions for occupational health staff in the past. The control group included information on effective smoking cessation strategies such as increasing the salience of information, equivalent to the Attractive nudge [23]. However, it is highly likely that it was not read due to information overload. In promotions, business-to-business (targeting for the first customer) is important in addition to business-to-customer (targeting for the final customer) [24]. When creating flyers on smoking, we tend to focus on smokers as the final customers but should not belittle the first customers. Actually, the primary competitor for a smoking cessation program is the tobacco industry, which actively engages in business-to-business marketing as the distribution strategy [25]. In contrast, the flyer in the control group failed to achieve high satisfaction from the first customers, the occupational health staff. This suggests that a bottleneck may have occurred at the first customers’ phase, and the flyer may not have been effectively delivered to the final customers. Occupational health staff, frustrated by the powerful marketing strategies of the tobacco industry, may be overwhelmed by the information overload in flyers [26,27]. Consequently, Phase 1 should focus on resolving the bottlenecks encountered with existing flyers.

The flyer in the nudge group seemed to overcome the bottleneck; it was significantly more likely to be rated as "want to use," "want to recommend," and "read until the end" compared to the control group. Since there were no items that stood out as highly rated in each nudge element, it is natural to interpret this as a synergistic effect of combining multiple nudge elements. Generally, incorporating all elements of the EAST may compromise the Easy element, but this study did not do so [28]. This combination can be a reference for future promotions. Alternatively, considering that the flyers for the control group received negative evaluations due to information overload, occupational health staff who are daily exposed to numerous health promotion flyers might have responded positively to those with reduced inhibiting factors [16]. The results of this study suggest that by focusing and minimizing the information presented in flyers and integrating the EAST framework, it is possible to resolve the bottlenecks in phase 1 and enhance business-to-business strategies.

There was no significant difference in the main outcome of “Willingness to apply.” Workers desiring smoking cessation tended to give favorable ratings to the flyer in the nudge group, similar to occupational health staff, as expected. However, contrary to our expectations, some items in the control group’s flyer received moderately favorable ratings. One possible explanation for this is the mere-exposure effect, as the control group’s flyer was a typical smoking cessation flyer and familiar to participants [29]. Nevertheless, there were no comments in the free-text responses supporting the occurrence of the mere-exposure effect, and this study could not identify the reasons why workers desiring smoking cessation evaluated the control group flyer positively.

Several items such as those in the nudge group received significantly higher ratings than those in the control group, such as readability, ease of application, and completion rates. One reason for higher ratings of these items could be that the nudge group removed no-smoking illustrations and expressions that completely negate smokers' values, such as "smoking is with all pain, no gain," and also removed emphasized expressions that smoking is annoying. Of note, these designs are commonly observed in flyers for smoking cessation programs. However, for smokers, who showed an automatic approach bias, no-smoking illustrations may evoke disgust emotions [30]. Expressions that negate smokers' values may also be perceived as disgusting by smokers who exhibit psychological reactance [31]. Emphasizing that smoking is annoying may lead them to feel shame or guilt. The expressions to evoke emotions like disgust, shame, and guilt may lead to underestimating health risks and could be inhibiting factors for ease of application or losing motivation to read it right away [32]. Therefore, it is suggested that typical expressions found in smoking cessation flyers might be better if omitted. Furthermore, the phrase in the control group’s flyer, "our company’s smoking rate is high," might inhibit interest in applying for the program because smokers tend to exhibit peer bias [33,34]. Removing the expressions from the control group's flyer and instead prominently displaying "69.1% succeeded in smoking cessation/reduction" in the nudge group likely contributed to the high ratings. The findings suggest that there is a need to design Social nudges that encourage smoking cessation.

Although the above suggested the impact of individual nudges, relying solely on nudges may be insufficient to increase the main outcome significantly. Considering the report that the most effective interventions involve combining different elements and that incentives have been reported to aid smoking cessation, it may be necessary to combine nudges with incentives to further increase the application motivation of workers desiring smoking cessation [35-37]. On the other hand, due to the cost issues associated with providing incentives, phase 2 will likely require the design of flyers that use nudges to enhance the salience of limited financial resources for incentives, thereby increasing their effectiveness [23].

This study has several limitations. First, there was a difference in the recruitment methods for occupational health staff and workers desiring smoking cessation. Second, the survey was conducted online, and particularly for occupational health staff, it targeted participants in smoking cessation seminars, potentially leading to selection bias. Third, the promotion of smoking cessation programs is done using paper in some workplaces. Therefore, caution is needed in interpreting the results of this study. Fourth, double-blinding was not conducted. However, the outcomes were measured objectively and minimized the risk of observer bias, thereby likely averting significant issues. Finally, the purpose of this study was to understand changes in motivation, and it does not address the adoption of the nudge group's flyer by occupational health staff, the application of workers desiring smoking cessation, or the extent to which the smoking cessation rate increased. Future long-term verification through intervention studies is necessary.

## Conclusions

A randomized controlled trial was conducted using a web survey to compare the evaluation of smoking cessation program flyers using nudges with typical flyers among occupational health staff and smokers. Occupational health staff did not favor typical flyers, but combining EAST nudges suggested a potential for them to actively promote the program. Workers desiring smoking cessation also showed a tendency to prefer the nudged flyers. To further enhance their motivation to apply for the program, it may be necessary to introduce other methods such as incentives.
